# A Critical Analysis of the Automated Hematology Assessment in Pregnant Women at Low and at High Altitude: Association between Red Blood Cells, Platelet Parameters, and Iron Status

**DOI:** 10.3390/life12050727

**Published:** 2022-05-13

**Authors:** Ramón Figueroa-Mujica, Luis Angel Ccahuantico, Maycol Suker Ccorahua-Rios, Juan José Sanchez-Huaman, Cinthya Vásquez-Velasquez, Jorge M. Ponce-Huarancca, Rodrigo E. Rozas-Gamarra, Gustavo F. Gonzales

**Affiliations:** 1 Escuela Profesional de Medicina Humana, Facultad de Ciencias de la Salud, Universidad Nacional de San Antonio Abad del Cusco, Cusco 08002, Peru; ramon.figueroa@unsaac.edu.pe (R.F.-M.); lccahuantico@gmail.com (L.A.C.); maycol.s.c.r@hotmail.com (M.S.C.-R.); mh.juanjosesh@gmail.com (J.J.S.-H.); engelsyk_mj@hotmail.com (J.M.P.-H.); rdrg18100@gmail.com (R.E.R.-G.); 2 Laboratorio de Endocrinología y Reproducción, Laboratorio de Investigación y Desarrollo (LID), Facultad de Ciencias y Filosofía, Universidad Peruana Cayetano Heredia, Lima 15102, Peru; cinthya.vasquez.v@upch.pe; 3 Departamento de Ciencias Biológicas y Fisiológicas, Facultad de Ciencias y Filosofía, Universidad Peruana Cayetano Heredia, Lima 15102, Peru; 4 Instituto de Investigaciones de la Altura, Universidad Peruana Cayetano Heredia, Lima 15102, Peru

**Keywords:** hematology, high altitude, iron status, erythropoietic hormones, hepcidin, IL-6

## Abstract

The objectives of the study were to determine differences in the parameters of red blood cells (RBC), white blood cells (WBC), and platelets at low altitude (LA) and at high altitude (HA) and with the gestation being advanced, and to determine correlations between parameters of RBC and platelets. We also studied the association of RBC and platelets with markers of iron status. In addition, markers of iron status and inflammation were measured and compared at each trimester of gestation in pregnant women at LA and HA. A cross-sectional comparative study was conducted at Lima (150 m above sea level) and Cusco at 3400 m above sea level from May to December 2019. Hematological parameters in pregnant women (233 at LA and 211 at HA) were analyzed using an automated hematology analyzer. Serum ferritin levels, soluble transferrin receptor (sTfR), hepcidin, erythropoietin, testosterone, estradiol, and interleukin-6 (IL6) levels were measured by ELISA. One-way ANOVA supplemented with post hoc test, chi-square test, and Pearson correlation test statistical analyses were performed. *p* < 0.05 was considered significant. Pregnant woman at HA compared to LA had significantly lower WBC (*p* < 0.01), associated with higher parameters of the RBC, except for the mean corpuscular volume (MCV) that was no different (*p* > 0.05). Platelets and mean platelet volume (MPV) were higher (*p* < 0.01), and platelet distribution width (PDW) was lower at HA than at LA (*p* < 0.01). A higher value of serum ferritin (*p* < 0.01), testosterone (*p* < 0.05), and hepcidin (*p* < 0.01) was observed at HA, while the concentration of sTfR was lower at HA than at LA (*p* < 0.01). At LA, neutrophils increased in the third trimester (*p* < 0.05). RBC parameters decreased with the progress of the gestation, except RDW-CV, which increased. The platelet count decreased and the MPV and PDW were significantly higher in the third trimester. Serum ferritin, hepcidin, and serum testosterone decreased, while sTfR and serum estradiol increased during gestation. At HA, the WBC and red blood cell distribution width- coefficient of variation (RDW-CV), PCT, and serum IL-6 did not change with gestational trimesters. RBC, hemoglobin (Hb), hematocrit (Hct), mean corpuscular hemoglobin concentration (MCHC), and platelet count were lower as gestation advanced. MCV, MPV, and PDW increased in the third trimester. Serum ferritin, testosterone, and hepcidin were lower in the third trimester. Serum estradiol, erythropoietin, and sTfR increased as gestation progressed. Direct or inverse correlations were observed between RBC and platelet parameters and LA and HA. A better number of significant correlations were observed at HA. Hb, Hct, and RDW-CV showed a significant correlation with serum ferritin at LA and HA. Of these parameters, RDW-CV and PDW showed an inversely significant association with ferritin (*p* < 0.05). In conclusion, a different pattern was observed in hematological markers as well as in iron status markers between pregnant women at LA and HA. In pregnant women a significant correlation between several RBC parameters with platelet marker parameters was also observed. Data suggest that pregnant women at HA have adequate iron status during pregnancy as reflected by higher serum ferritin levels, lower sTfR levels, and higher hepcidin values than pregnant women at LA.

## 1. Introduction

Reports to date estimate that over 100 million people permanently reside at altitudes above 2500 m [1]. Peru has 30 million inhabitants of which 10 million people live above 2000 m of altitude [2].

Permanent life at high altitude (HA) induces important physiological stresses linked to exposure to chronic hypoxia. Various strategies have been adopted by diverse populations living in the Andes, Tibet, and East Africa. The main mechanism is an increase in red blood cell production, more marked in Andeans than in Tibetans or Ethiopians. Other changes are observed in the cardiovascular or respiratory systems, as well as in the utero-placental circulation [3]. Erythrocytosis, or increased production of red blood cells, is one of the most well-documented physiological traits that varies within and among HA populations [4].

Pregnancy is a situation that could show different patterns at low altitudes (LA) and also at HA. Physiological changes during pregnancy concerning non-pregnant women are necessary to ensure an adequate nutritional supply and the development of the fetus [5]. During pregnancy, physiologic iron demands a substantial increase by one gram to support fetoplacental development, maternal adaptation to pregnancy, and parturition [6]. Red blood cells (RBC) during pregnancy tend to increase in number; however, blood plasma increases to a greater extent, generating blood hemodilution that also affects platelets as pregnancy progresses, causing a slight progressive decrease in the concentration of both cells [7,8]. Serum ferritin also decreases as an effect of hemodilution but also may reflect efficient iron mobilization from stores in agreement with the progressive hepcidin decrease during pregnancy [6].

In healthy pregnancies, plasma volume begins to expand in the first trimester, has the steepest rate of increase in the second trimester, and peaks late in the third trimester [9]. During physiological pregnancy, plasma volume increases by, on average, more than 1 L as compared with non-pregnant conditions. In pregnancies complicated by pregnancy-induced hypertension, preeclampsia, or fetal growth restriction, plasma volume increase in the third trimester is 13.3% lower than in normal pregnancy, and this may result in hemoconcentration [10].

Both cell lines, platelets, and RBC share characteristics such as being anucleate cells and having the same origin through myeloid progenitors [11]. Although a relationship between RBC parameters and platelet parameters has been described at LA [11], no study has been performed at HA.

Anemia during pregnancy is considered an adverse condition that can affect the health of the maternal–fetal unit according to the World Health Organization (WHO), generally defined with hemoglobin values <11 g/dL. Several studies have demonstrated that hemoglobin values in pregnant women between 9 g/dL and 13 g/dL are not associated with adverse neonatal outcomes [12,13,14,15]. This is an important question for populations living at HA since they have normally increased Hb levels [2].

Life at HA has been associated with decreased uterine artery blood flow, increased utero-placental resistance, alterations in the expression of placental factors, chronic hypoxia, and changes in vascularity and has been implicated in a variety of adverse pregnancy outcomes including intrauterine growth restriction, low birth weight infants, intrauterine fetal demise, and preeclampsia [16]. It is assumed that high Hb at HA is not associated with iron deficiency (ID) in adult men and women [17]; however, no studies have been conducted to evaluate serum ferritin, serum transferrin receptor (sTfR), and serum hepcidin levels during pregnancy at HA.

The advent of the automatic hematology analyzer has allowed numerous parameters of RBC, white blood cells (WBC), and platelets to be analyzed simultaneously. We have assessed in the present study, blood samples from pregnant women residing at two altitude levels, one at an LA of 150 m above sea level (m.a.s.l) and the second at an HA of 3400 m.a.s.l. The objectives of the study were to determine differences in the parameters of RBC, WBC, and platelets at LA and at HA and the gestation being advanced, and to determine correlations between parameters of RBC and platelets. RBC and platelet counts have been associated with iron status [18], but both are also associated with hemodilution. For such a reason, we studied the association of RBC and platelets with markers of iron status. In addition, markers of iron status and inflammation were measured and compared at each trimester of gestation in pregnant women at LA and HA. At the same time, hormones regulating erythropoiesis such as erythropoietin, testosterone, and estradiol were assessed in this study.

## 2. Materials and Methods

### 2.1. Design

A total of 444 pregnant women (first, second, and third trimester) aged 18–45 years from Lima at 150 m above sea level with an environmental pressure of 150 mm Hg (N = 233) and Cusco at 3400 m above sea level (N = 211) with an environmental pressure of 513 mm Hg were included under a comparative cross-sectional study design and convenience sampling technique.

### 2.2. Sample Size Determination

The sample size was determined by considering the following assumptions: α (two-sided) = 0.05; power = 0.95; effect size = 0.4, and the sample size required was a minimum of 164 pregnant women at each place of study [19]. These pregnant women were allocated into three groups (first trimester, second trimester, and third trimester) at each place of study. In Lima 31 women in the first trimester, 52 in the second trimester, and 149 in the third trimester were studied. In Cusco 58 women in the first trimester, 81 in the second trimester, and 69 in the third trimester were studied. The first trimester was defined when gestational age was ≤12 weeks; the second trimester defined when gestational age was 13–26 weeks; the third trimester when gestational age was 27–40+ weeks. The final sample included 233 pregnant women at LA and 211 pregnant women at HA.

The inclusion criterion was residence in the city of Lima (150 m.a.s.l) or Cusco (3400 m.a.s.l) for the last 10 years. The exclusion criteria were chronic smokers, pregnant women with medical treatment in the last three months for metabolic or hematological disorders, pregnant women with treatments for mental disorders, and pregnant women with multiple pregnancies. During the period of the study, no pregnant women had a history of chronic smoking. No pregnant woman included in the study had low-dose aspirin intake.

### 2.3. Enrolment and Variables Measured

After signing the informed consent form, physiological data were obtained from each eligible pregnant woman. Pre-pregnancy weight was recorded. BMI was calculated by dividing pre-pregnancy body weight in kilograms over height in meters squared (Kg/m^2^). Finger index pulse oxygen saturation (SpO_2_) and systolic and diastolic blood pressure (SBP and DBP, mm Hg) were measured in a sitting position before blood sampling. SpO_2_ was measured as % on the index finger by the pulse-oximeter (Riester 1905 RI-fox N). Blood pressure was measured in the volunteer’s left arm, in a sitting and comfortable position, using an aneroid sphygmomanometer. Arterial oxygen content (CaO_2_) was calculated using SpO_2_ and Hb data with the formula: CaO_2_ (mL O_2_/dL) = (1.34 × Hb × Sat. O_2_/100) + (0.003 × PaO_2_), where the maximum volume of oxygen that combines with 1 g of hemoglobin is 1.34 [20,21]. Arterial oxygen pressure (PaO_2_) for LA = 85.5 mm Hg and PaO_2_ for pregnant women at HA = 65.2 mm Hg [22].

The participants voluntarily filled out a sociodemographic questionnaire related to their place of birth, occupation, mother tongue, education level, and civil status.

### 2.4. Sample Collection

Venous blood samples collected in the first, second, or third trimester using standard procedures were placed in three tubes. One containing spray-coated K_2_EDTA (Becton Dickinson Medical Devices Co Ltd., Frankin Lakes, NJ, USA) and in two tubes without additives to obtain serum. Nine milliliters of venous blood were collected in the fasting state.

### 2.5. Automated Blood Count

For the automated blood count, the 25-parameter CELL-DYN Ruby^®^ automatic multi-analyzer was used at each place of the study. This hematological analyzer is based on the optical laser detection method using MAPSS technology (Multi-Angle Polarized Scatter Separation). Complete blood count included red blood cell count (RBC), hemoglobin (Hb), hematocrit (Hct), mean corpuscular volume (MCV), mean corpuscular hemoglobin (MCH), mean corpuscular hemoglobin concentration (MCHC), red blood cell distribution width-coefficient of variation (RDW-CV), platelet count (PLT), mean platelet volume (MPV), platelet distribution width (PDW), plateletcrit (PTC), white blood cell count (WBC), and leukocyte differential count (neutrophil, lymphocytes, monocyte, eosinophils, and basophils). The hematology analyzer was calibrated and maintained according to the instruction of the manufacturer. Three levels of quality control were made twice every day to ensure good precision. Completed blood count was performed at each place (Cusco and Lima). Hematological markers were measured within 2 h after blood drawing in Lima and Cusco.

### 2.6. Laboratory Analysis

Commercial enzyme-linked immunoassay kits (DRG, GmBh, Marburg, Germany) were used to quantify hepcidin (ng/mL), ferritin (ng/mL), soluble transferrin receptor (ug/mL), erythropoietin (mU/mL), testosterone (ng/mL), estradiol (pg/mL), and interleukin-6 (pg/mL). Measurements were performed in serum sample kept frozen (−40 °C) before being assessed. All assessments were conducted in the laboratory in Lima.

### 2.7. Ethical Aspects

Data for the present study were obtained with prior authorization from the Direction of the Regional Hospital of Cusco—Ethics and Research Committee and the Regional Health Direction of Cusco and from the Direction of the Instituto Nacional Materno-Perinatal (National Maternal-Perinatal Institute) in Lima, Peru. Pregnant women who met the inclusion and exclusion criteria were invited to participate in the study and sign the corresponding informed consent. The identification of the volunteers was protected by codes; in addition to this, they knew about each of the tests that was to be carried out, as well as the objectives of the study. To proceed with the study and sampling, the volunteers signed informed consent. The study was conducted following the declaration of Helsinki.

### 2.8. Statistical Analysis

The study is cross-sectional, analytical, and prospective. Analyses were conducted using the STATA v16.0 statistical package (StataCorp., College Station, TX, USA). The univariate analysis of the sociodemographic variables of the pregnant women was carried out using the absolute frequencies and statistically assessed using the chi-square test for the qualitative variables and the means ± standard error of the mean for the quantitative variables, for the overall pregnant women at each altitude, and then for each trimester of gestation. After confirmation of normality for each continuous variable using the Shapiro–Wilk test and homogeneity of variances with Bartlett test, quantitative data were compared between HA and LA by independent “*t*” test. Differences between trimesters of pregnancy were performed using the one-way analysis of variance (ANOVA) test, and if they presented a significant difference, Scheffé’s post hoc multiple comparison test was used to determine the difference between pairs of means. Quantitative data of all study groups were compared for statistical significance. The association of seven red blood cell parameters with four platelet parameters was performed mostly by the Pearson correlation coefficient (r), with a significance level of *p* < 0.05 and eventually by polynomial or power correlation. Finally, for the association of RBC and platelet parameters with the serum ferritin level, the Pearson correlation coefficient (r) was also used. A *p*-value of ≤0.05 denotes statistical significance.

## 3. Results

Table 1 presents the characteristics evaluated in the population of pregnant women at LA (Lima, 150 m) compared with those observed at HA (Cusco, 3400 m). The analysis of the quantitative variables shows that at HA as opposed to LA, the pregnant women had a significantly younger age, lower gestational age at the time of evaluation, lower value of pulse oxygen saturation (SpO_2_), higher arterial oxygen content (CaO_2_), and lower value of systolic (SBP) and diastolic blood pressure (DBP). Pregnant women residing at HA had as an antecedent a lower number of children born alive (<0.01), whereas the number of stillbirths was similar at LA and HA (*p* > 0.05).

Alcohol consumption and smoking were negligible at both LA and HA (*p* > 0.05). The analysis of the qualitative variables shows that, at LA, 74.5% of the pregnant women were born in LA areas (coast or jungle), while, of the pregnant women residing at HA, in 99% of cases they were born in highland areas (chi-square test: 242; *p* < 0.01).

The educational level was similar in pregnant women at LA and HA (chi-square test: 0.11; *p* > 0.05). Most pregnant women in both LA and HA had a high school education (59% and 58%, respectively). At LA, 35.5% of pregnant women had studied at university with little difference to the pregnant women at HA (37%; *p* > 0.05). The most predominant civil status was that of unmarried partners, being greater in pregnant women at HA than in those at LA. The highest number of married pregnant women was observed at LA (chi-square test: 41.95; *p* < 0.01). No differences in occupational level were observed between pregnant women residing at LA and HA (chi-square test: 1.89: *p* > 0.05). A greater percentage of the pregnant women were housewives (58.7% in LA and 60.2% in HA).

The automated hematological analysis showed that the pregnant woman at HA compared to a woman at LA had significantly lower counts of WBC, neutrophils, and monocytes (*p* < 0.01), while in the blood-red series a higher count of RBC, a higher concentration of Hb, higher hematocrit, greater MCH, and MCHC were observed (*p* < 0.01). The mean corpuscular volume (MCV) was no different in LA and HA pregnant women (*p* > 0.05). Platelets, mean platelet volume (MPV), and plateletcrit (PCT) were higher in pregnant women at HA (*p* < 0.01). RDW-CV and PDW were both lower at HA than at LA (*p* < 0.01).

With regards to the biochemical and hormonal analysis, a higher value of serum ferritin (*p* < 0.01), testosterone (*p* < 0.05), and hepcidin (*p* < 0.01) was observed at HA, while the concentration of the sTfR was lower at HA than at LA (*p* < 0.01). 

In LA pregnant women, the arterial oxygen content (CaO_2_) decreased with the advance of gestation. Neutrophils increased and lymphocytes and eosinophils decreased in the third trimester (*p* < 0.05). RBC, Hb, and Hct decreased in the second trimester of pregnancy and recovered in the third trimester compared to the second trimester, although their mean value in the third trimester continued to be lower than in the first trimester (*p* < 0.01). MCH was unchanged but MCHC was lower in the third trimester, while RDW-CV increased as gestation progressed, being higher in the third trimester compared to the second and first trimesters of gestation (Table 2).

The platelet count decreased in the second trimester and remained at those values in the third trimester. The MPV was significantly higher in the third trimester than in the first trimester; PDW increased in the third trimester compared to the value of the first and second trimesters (Table 2).

In relation to biochemical and hormonal markers, serum ferritin and serum testosterone decreased, while serum estradiol increased in the third trimester compared to the values of the first and second trimester. sTfR was higher in the third trimester compared to the first trimester of gestation (*p* < 0.01). Serum hepcidin was lower in the second and decreased more in the third trimester compared to the values of the first trimester (Table 2).

Table 3 presents the results by trimester in pregnant women at HA. The pulse oxygen saturation (SpO_2_) was lower in the third than in the second trimester (*p* < 0.05). SBP and DBP increased in the third trimester. The arterial concentration of oxygen (CaO_2_) was lower in the third trimester than in the second (*p* < 0.01) and first (*p* < 0.01) trimesters of gestation.

The WBC count, neutrophils, monocyte, lymphocytes, and basophils did not change with gestational trimesters. Eosinophils were lower in the third trimester than in the second (*p* < 0.05) and first (*p* < 0.01) trimesters.

RBC, Hb, and Hct were lower in the second and third trimesters compared to values in the first trimester. From the second to the third trimester of pregnancy, the values remained unchanged (*p* > 0.05). MCV increased in the second and third trimesters compared to values in the first trimester. MCH was maintained as unchanged, whereas MCHC decreased in the third trimester compared to values in the first trimester (<0.05). RDW-CV did not change in the three trimesters of gestation (*p* > 0.05).

The platelet count decreased, while MPV and PDW increased in the third trimester compared to the second and first trimesters. The values in the first and second trimesters remained unchanged (*p* > 0.05). PCT was not modified in any of the three trimesters of gestation (*p* > 0.05).

All biochemical and hormonal markers changed over the course of gestation except for the concentration of IL-6 which remained unchanged (*p* > 0.05). Serum ferritin was lower in the second trimester than in the first trimester and was reduced further in the third trimester compared to the second trimester (*p* < 0.05).

Serum testosterone was lower in the third trimester compared to the first and second trimesters, with no change in its values between the first and second trimester. Serum estradiol increased in the second trimester (*p* < 0.01) and even more so in the third trimester of gestation (*p* < 0.05). Serum EPO was higher in the second trimester (*p* < 0.01) and in the third trimester (*p* < 0.05) compared to the values of the first trimester of pregnancy. The serum EPO values of the second and third trimesters were no different (*p* > 0.05).

sTfR was higher in the third trimester than in the second (*p* < 0.05) or in the first trimester (*p* < 0.05). sTfR values were not different between the first and second trimesters (*p* > 0.05). Serum hepcidin was lower in the second and third trimesters compared to the values in the first trimester. There were no differences in the hepcidin values of the second and third trimesters (*p* > 0.05).

Pearson correlation coefficients value (r) for seven RBC parameters and four platelet parameters at LA and at HA can be observed in Table 4. At LA, a significant direct correlation was observed between RDW-CV and platelet count and between RBC count and PCT. A significant inverse correlation was observed between MCV or MCH with platelet count and MCH. MCHC had an inverse correlation with PCT. In summary, of 28 correlations, seven were statistically significant (five with a negative correlation and two with a positive correlation). At HA, a significant direct correlation was observed between RBC count or RDW-CV with platelet count or with PCT. Different from the situation at LA, pregnant women at HA showed a direct correlation of Hb, Hct, MCV, and MCH with PDW. MCV, MCH, and MCHC correlated inversely with platelet count and PCT. RDW-CV correlated negatively with PDW. In summary, from 28 correlations, 15 were statistically significant (seven with a negative correlation and eight with a positive correlation). The MPV values showed no correlation with the seven parameters of RBC in pregnant women at LA and at HA (*p* > 0.05).

When correlating gestational age with hematological parameters of the red series, it is observed that for both pregnant women resident at LA and those residing at HA, the RBC count, Hb concentration, Hct, and MCHC were inversely correlated with gestational age (*p* < 0.05). CaO_2_ was also inversely correlated with gestational age at both LA and HA. Among the markers of iron status, serum ferritin and hepcidin showed an inverse and statistically significant correlation with gestational age in both LA and HA populations (Table 5).

MCV showed a significant direct correlation with gestational age at high altitude but not at low altitude. MCH showed an inverse and weak significant correlation with gestational age at LA but not at HA. sTfR and EPO did not change with gestational age at LA but showed a direct and significant linear relationship with gestational age at HA (Table 5). At LA, further analysis showed that the correlation was nonlinear (polynomic correlation; r = 0.15; *p* < 0.05).

After correlating seven red blood cell parameters with serum ferritin levels, three hematological parameters (Hb, Hct, and RDW-CV) showed significant correlation in both populations (LA and HA). Of these parameters, only RDW-CV showed an inversely significant association (*p* < 0.05). RBC correlated significantly at HA but not at LA; MCH correlated significantly at LA but not at HA; MCHC significantly correlated at HA but not at LA. MCV did not correlate with serum ferritin at either LA or HA.

By correlating four platelet parameters with serum ferritin levels at LA and HA, it is observed that the only significant and inversely proportional correlation was observed between PDW and serum ferritin at both LA and HA (Table 6). Platelet count, MPV, and PCT were not significantly associated with serum ferritin levels.

## 4. Discussion

The present study was designed to compare different physiological, hematological, and iron marker parameters in pregnant women from two populations in Peru, one resident at LA and another at HA. 

It is confirmed that pulse oxygen saturation (SpO_2_) is lower in pregnant women at HA than at LA [23]. In addition, we observed that SpO_2_ is reduced as gestation progresses in residents at HA compared with those residents at LA. These results confirm data in Ethiopia in a resident population at 2840 m of altitude [24], and in Bolivia at 3600 m [25], where SpO_2_ values were reduced as gestation advanced, while at LA, SpO_2_ did not change throughout gestation [26].

Compared to non-pregnant women, SpO_2_ increased with gestation at 3600 m [25]. In Cusco at 3400 m, the mean SpO_2_ in non-pregnant women was 91.06% [17], a value that is lower than that observed in pregnant women living in the same place (94.91%), while the average value of SpO_2_ at 150 m in non-pregnant women was 98.41%, which did not vary later during pregnancy.

Hb concentration and arterial oxygen content (CaO_2_) were higher in pregnant women at HA than at LA. At 3400 m, the SpO_2_ and Hb concentration decreased towards term, resulting in a fall in CaO_2_ at the end of pregnancy as observed at 4330 m [23].

Systolic (SBP) and diastolic blood pressure (DBP) remained unchanged at LA according to the progress of pregnancy, whereas at HA, the SBP and DBP values were lower than at LA but increased in the third trimester compared to values in the first or second trimesters. This increase in the values of SBP and DBP with the advance of pregnancy at 3400 m compared to those observed at 150 m may be associated with the greater description of higher blood pressures throughout pregnancy and preeclampsia at altitude [27]. A systematic review and meta-analysis showed that maternal blood pressure is higher at term in pregnancies at HA than in LA, accompanied with an increased risk of gestational hypertension but not preeclampsia [28].

The reduced SpO_2_ in pregnant women at HA might be due to diminished partial pressure of oxygen, which reduces alveolar oxygen tension, but also to hemodilution that could be higher at HA than at LA [24]. This can be verified with the degree of reduction in Hb from the first to the third trimester, of 5.6% at LA and 6.5% at HA, and from the first to the second trimester of 6.8% at LA and from 7.3% at HA. The higher arterial oxygen content (CaO_2_) at 3400 m suggests that a deficit of oxygen availability at the tissue level is not occurring; even more so in the three trimesters of gestation, the CaO_2_ was greater at HA than at LA. The plasma volume was not measured in each trimester of gestation, and this is a limitation of the current study.

The WBCs were at lower values at HA and among them there was a significant decrease in neutrophils and monocytes. Studies at an altitude of 2270 m.a.s.l have also shown lower values of neutrophils than at sea level [29], confirming our results at 3400 m.a.s.l.

In a normal LA pregnancy in Morocco, the WBCs increased significantly from the first to the third trimester. Moreover, total WBC and the mean value of the number of neutrophils were significantly higher for pregnant women compared with non-pregnant women. In addition, a significantly progressive increase in neutrophils according to the gestational age was observed [30]. At LA, our study shows that pregnant women have higher neutrophil counts as gestation progresses from the first to the third trimester. This pattern was not observed in pregnant women at HA. To our knowledge, this is the first report on neutrophil assessment in pregnant women living at HA and the demonstration of the absence of changes as pregnancy progresses.

It is assumed that elevated leukocyte levels associated with pregnancy at LA are due to the physiological stress led by the state of pregnancy, and an increase in the number of neutrophils is probably a homeostatic answer to apoptosis of the altered neutrophil expressed during pregnancy [31]. The finding that IL-6 (a pro-inflammatory cytokine) levels were not different between pregnant women at LA and in HA but that their values were unchanged during gestation, suggests that differences in WBC during normal pregnancy are not associated with inflammatory markers. 

Neonatal birth weight was positively correlated with circulating WBC in the third trimester [32]. Although there are no studies that associate the count of WBC and neutrophils with birth weight at HA, our results of a lower count of WBC and neutrophils in pregnant women of HA and their absence of increase in both hematological markers throughout gestation would explain the lower birth weight observed in HA populations. A recent systematic review, meta-analysis, and meta-regression showed globally increases in the likelihood of adverse perinatal outcomes, including low birth weight (LBW), small-for-gestational-age (SGA), and spontaneous preterm births (sPTB), in HA pregnancies. There is an inverse relationship between birth weight and altitude [33]. 

Data from the current study show that pregnant women at HA in addition to lower WBC counts have higher RBC and platelet values than at LA (150 m). It is known that RBC, Hb, and Hct increase with altitude more in normal adult men and women with shorter time of residence than in those with higher generational time of residence considered as adapted to live at high altitudes [34,35], and this pattern is observed also during pregnancy [36,37].

At LA and at HA, the RBC, Hb, and Hct values declined as gestation progressed, reaching their lowest mean value in the second trimester, and began to rise again in the third trimester. A similar pattern has been described in another study at LA [38]. 

The reduction in these hematological markers has been associated with a normal hemodilution due to the higher increase in plasma volume related to the increase in the red mass. This results in a reduction in Hb concentration during pregnancy to values below the cut-off point of 11 g/dL of normality defined by the World Health Organization as observed in a study in non-anemic pregnant women in Turkey. This study showed that the lower reference value for Hb was calculated as 10.67, 10.08, and 9.18 g/dL for 10–14, 20–24, and 30–34 gestational weeks, respectively. The authors suggest that iron supplementation may not be needed as any decrease is due to physiological hemodilution. These results may prevent unnecessary iron prescription during pregnancy [39]. As Hb values change with the trimester of gestation, the need for iron supplements is different in each trimester.

Our study also confirms that the population of pregnant women living at HA has better iron status than those at LA, demonstrated as high serum ferritin levels, low sTfR levels, and high hepcidin levels observed in pregnant women at HA than at LA.

MCV values were similar in pregnant women at LA and HA. In our study, the mean values were 92.22 fL in pregnant women at LA and 92.92 fL at HA. These values are considered normal and are not associated with any tendency to iron deficiency anemia (IDA) [40], even more so considering that Hb values were on average 11.67 ng/dL at low altitude and 14.44 g/dL at HA. In the present study, MCV was unchanged from the first to the third trimester at LA, but it was increased from the first to the third trimester at HA. Values of MCV < 86 fL have been defined as being associated with iron deficiency anemia [41]. This is an important finding because it suggests that ID is not a problem at HA as has been suggested previously [36]. For such a reason, a correction of the Hb cut-off point to define anemia in pregnant women at high altitudes could be unnecessary.

Normal values for MCH and MCHC in pregnant women at LA and at HA also suggest that the probability for IDA at HA is lower. This is further confirmed with higher serum ferritin values at HA than at LA, despite the fact that serum IL-6, an inflammatory marker, is not different at both altitudes. Similarly, maintaining similar MCH values throughout pregnancy at HA suggests that no criterion leads to an increase in the risk of true anemia and even more of IDA.

During normal pregnancy, RDW-CV values progressively increase [42,43]. This finding has been confirmed in our study at LA. However, the pattern in pregnant women at HA was different since RDW-CV remained unchanged throughout pregnancy.

By contrast, a previous study showed that the values of RDW, platelet count, mean platelet volume, platelet distribution width, and plateletcrit did not show any significant differences at the different trimesters of pregnancy in apparently healthy pregnant subjects in Port Harcourt, southeastern Nigeria [44]. The authors suggested that increases in unsaturated and total iron-binding capacity and serum transferrin values seen among pregnant women with increasing gestation may be a mechanism to ensure a fetal adequate iron delivery. Our study suggests that iron status is better at HA than at LA since serum ferritin and hepcidin levels were higher, whereas sTfR levels were lower than at LA. In both cases, EPO levels were not different (Table 1).

Platelets have been described to be higher in people with a long time of residence in Tibet with respect to values observed in the Han population residing for a shorter generational time in Tibet [34]. A systematic analysis study has shown that the platelet counts of a chronic mountain sickness group were lower compared with the healthy altitude control group [45]. In addition, the observation that the platelet count is higher in pregnant women at HA than at LA is consistent with the suggestion that populations living in the Southern Andes in Peru are more adapted than those from the central Andes [46]. These data suggest that adapted people (with higher generational time residing at high altitudes) have, compared to sea level counterparts or non-adapted HA residents, less Hb concentration and higher platelet counts. 

The differences in hematological parameters between different populations living at HA have been assessed and confirmed [47]. 

During human pregnancy, maternal platelet count decreases gradually from the first to the second and third trimesters. In addition to hemodilution, accelerated platelet sequestration and consumption in the placental circulation may contribute to a decline in platelet count throughout gestation [8]. RBC, Hb, and Hct are also reduced during pregnancy as an effect of normal hemodilution due to maternal blood volume expansion occurring at a larger proportion than the increase in red blood cell mass [5]. 

Platelet count was significantly lower in a preeclamptic group compared to that in a control group, whereas MPV and PDW were significantly higher in women with preeclampsia [48]. Platelet count had a significant but negative correlation with the duration of hypertension illness, while MPV showed a positive and significant correlation [49]. Increased platelet activation, and the subsequent occurrence of placental fibrinoid deposition, are linked to placenta pathologies such as preeclampsia [50].

It is known that erythropoiesis and thrombopoiesis are processes with a common stem cell, the colony forming unit-erythrocyte megakaryocyte (CFU-EMk). An association between markers of RBC and markers of megakaryocytes has been described [11]; however, this is the first study approaching this association at LA and HA.

Despite these common pathways between the processes of erythropoiesis and thrombopoiesis, few studies have associated platelet parameters with those of the red cell series [11,18,51]. 

In the present study, we observed that pregnant women at LA have better correlations between platelet counts and plateletcrit with different parameters of the red cell series except for Hb and Hct. These findings are similar to those observed in 1250 men and women also using an automated hematology analyzer [11]. In pregnant women at HA, platelet counts and PTC have a similar correlation with that observed at LA; however, PDW, different from that observed at LA, has a significant correlation with Hb, Hct, MCV, MCH, and RDW-CV. These data suggest that life at HA modifies the association between RBC parameters and platelet parameters observed at LA. Further research would be necessary to determine the role of the correlations between RBC parameters and platelet parameters for the mother and the growing baby or if there is a link to pregnancy pathologies at LA and HA.

In pregnant women, Hb mass is increased and, due to this, an increase in EPO level is expected. A previous study showed that EPO concentration is almost 100% higher at the end of pregnancy [52]. In addition, in iron-repleted women, EPO increased less during pregnancy [53]. EPO level was best able to identify anemia and depleted iron stores [54]. This suggests that in an anemic condition, EPO will increase more than in pregnant women with normal iron status to increase erythropoiesis. We did not observe differences in EPO levels between pregnant women at LA, whereas at HA, EPO increased 28.6% from the first to the third trimester. The pregnancy-associated rise in EPO in pregnant women at HA relative to LA, despite a similar reduction in Hb from the first to the second trimester at LA and HA (7.3% and 7.8%, respectively), has been previously described, suggesting that an augmented pregnancy-associated increase in EPO levels may be important for successful vascular adaptation to pregnancy at HA [55].

A higher value in Hb concentration in pregnant women at HA compared with data at LA associated with similar values of serum EPO could be due to the high testosterone concentration observed in pregnant women at HA. High testosterone concentration has been associated with high Hb levels during pregnancy [56]. High testosterone levels have also been associated with high Hb levels in men at HA [57]. It is interesting to observe that serum testosterone is high in the first trimester but is reduced in the second and third trimesters at both altitudes. It is known that high maternal testosterone may have adverse neurodevelopmental outcomes [58,59] and lower offspring muscle strength [60]. For such a reason, a reduction in serum testosterone during pregnancy seems to be a way to reduce harmful effects on the offspring.

Estrogen levels are similar in LA and HA and increase as gestation progress, increasing much more in HA than in LA pregnant women. In the highland places of Bolivia, it has been shown that pregnant women with greater generational antiquity have higher levels of serum estradiol in pregnancy than those with less generational antiquity. In those with greater generational antiquity (more adapted), there is a greater increase in serum estradiol compared to what happens to those at LA [61].

Data on markers of iron status suggest that pregnant women at HA have better iron store and bioavailability than at LA. This is based on the fact that serum ferritin and hepcidin levels were higher and sTfR levels were lower at HA. The sTfR concentration reflects both the number of young erythrocytes and the degree of their iron deficiency. In pregnancy, sTfR concentrations do not seem to change compared with non-pregnant concentrations unless maternal erythropoiesis is iron deficient, and this should be associated with a reduction in serum ferritin levels. For such a reason, the sTfR concentration may only mildly increase by the third trimester in the iron-replete population but increase substantially in women with IDA [6]. 

Several studies have compared hematological parameters using automated blood counts with iron status. For example, an Indian study showed a negative correlation between RDW-CV and serum ferritin levels in pregnant women [62]. We also observed in our study that pregnant women at HA and LA have a negative correlation between RDW-CV and serum ferritin levels. These data suggest that higher RDW-CV and lower serum ferritin levels in pregnant women at LA than at HA mean that the probability of iron deficiency should be higher at LA than at HA.

In women with IDA, an inverse relationship has been observed between PDW and MCV [18], while at HA we observed a positive association between PDW and MCV, which possibly suggests that IDA is not a characteristic of the pregnant women studied here.

In IDA, decreased iron saturation might stimulate megakaryopoiesis. Moreover, iron may have an inhibitor effect on platelet counts [18]. In summary, we must expect to find IDA as pregnancy progresses as low levels of the iron store (low ferritin levels) are associated with low Hb and high platelet count. As observed in Table 3, both Hb and platelet counts are lowered as pregnancy advances.

In states of anemia due to chronic disease, reactive thrombocytosis occurs through cytokines such as IL-6 and thrombopoietin [63]. It is possible to affirm that ID in the bone marrow affects the conversion of cells into erythrocytes or platelets, where a context of ID favors the production of platelets [64]. In our study, we observed that during the progression of pregnancy, RBC and platelet counts decreased in a similar magnitude, suggesting that for the studied population, iron deficiency is not a problem. Moreover, IL-6 was similar at LA and HA, and its values were unchanged throughout gestation.

As PDW increases with gestational age and serum ferritin decreases with gestational age, the inverse relationship between PDW and serum ferritin is explained, but this is no evidence of any cause–effect association between these two variables. In a previous study, pregnant women with more severe and hypochromic anemia had higher PLT, PCT, and MPV [65]. In these cases, WBC, Hb, and Hct are reduced and associated with high platelet counts. We did not observe this association, suggesting that in the population studied here, the cases of severe and hypochromic anemia were few. 

To our knowledge, the mechanism of maternal hepcidin suppression during pregnancy is completely unknown. Plasma dilution may partially contribute, but the magnitude of hepcidin decrease cannot be explained by only a 30–50% increase in plasma volume. Moreover, plasma dilution would not explain the profound suppression of hepatic hepcidin messenger RNA that has been observed in animal studies [6]. The fact that serum hepcidin was higher at HA than at LA suggests that iron requirements are higher at LA than at HA. This is associated with higher serum ferritin levels in pregnant women at HA than at LA.

Lower hepcidin levels at HA may increase the Hb concentration (hemoconcentration) and this may pose adverse effects on the placental uterine flow. A recent study in Cusco (3400 m) showed that in a population of adult men and women living at high altitudes, the blood viscosity level is extremely high [66]. An increase in blood viscosity in a pregnant woman generates a decrease in placental uterine flow as has been demonstrated in the high Andean areas of Bolivia at 3600 m.a.s.l. 

Women with greater generational antiquity in the altitude of Bolivia have a greater uterine-placental flow and lower level of Hb than those pregnant women with less generational antiquity living in the same altitudinal zone [67]. Thus, pregnant women with a higher level of Hb have a lower uterus-placental flow and low birthweight children [68]. Therefore, the suggestion to modify the cut-off point of Hb to define anemia at HA may lead to the administration of iron that can in turn result in erythrocytosis, as has been demonstrated in populations of the altitude of Mexico, and this has been associated with an increase in oxidative stress [69] and children with low birth weight [70].

## 5. Conclusions

The results of this study suggest that a different pattern is observed in hematological as well as iron status markers in pregnant women at LA and at HA. In pregnant women a significant correlation between several RBC parameters with platelet marker parameters was also observed. These correlations are better observed at HA than at LA. In addition, data suggest that women at HA in Peru have adequate iron status during pregnancy as reflected by higher serum ferritin levels, lower sTfR levels, and higher hepcidin values than pregnant women at LA. Further research is required to consolidate these findings with pregnancy outcomes. Our data suggest that the cause of anemia in pregnant women should be adequately identified to allow the implementation of interventions, particularly with iron supplementation in women that really require it since it is effective in reducing anemia when administered in iron-replete mothers. Otherwise, iron supplementation could increase the risk of iron overload, hemoconcentration, and adverse pregnancy outcomes. 

## Figures and Tables

**Table 1:** Characteristics of pregnant women at low and at high altitude.

	Low Altitude (N = 233)	High Altitude (N = 211)
Age (years)	29.3 ± 0.47	27.30 ± 0.43 *
BMI (Kg/m^2^)	27.37 ± 0.65	26.78 ± 0.27
Gestational age (weeks)	27.37 ± 0.65	21.65 ± 0.72 *
Pulse oxygen saturation (%)	98.08 ± 0.05	94.91 ± 0.18 *
Arterial oxygen content (CaO_2_) (mL/dL)	12.60 ± 0.11	18.53 ± 0.10 *
Systolic blood pressure (mm Hg)	103 ± 0.74	98.73 ± 0.61 *
Diastolic blood pressure (mm Hg)	70 ± 0.62	63.12 ± 0.42
Drink alcohol	0/197 (0%)	0/210 (0%)
Smoke	1/197 (0.5%)	1/210 (0.47%)
Place of birth of pregnant women		
Low altitude	161 (75%)	2 (1%)
High altitude	55 (25%)	209 (99%) *
Marital status		
0. Single	46 (21%)	13 (6.3%) *
1. Married	48 (22%)	17 (8.2%)
2. Partner	124 (56.6%)	176 (85.4%)
3. Divorced	1 (0.4%)	0 (0%)
Number of live children	1.54 ± 0.03	0.76 ± 0.06 *
Stillbirths	0.03 ± 0.01	0.03 ± 0.01
Age at menarche (years)	12.60 ± 0.11	12.86 ± 0.19
WBC (10^3^/uL)	8.81 ± 0.14	7.79 ± 0.11 *
Neutrophils (10^3^/uL)	6.10 ± 0.11	5.50 ± 0.09 *
Lymphocytes (10^3^/uL)	1.91 ± 0.03	1.86 ± 0.03
Monocytes (10^3^/uL)	0.53 ± 0.01	0.22 ± 0.01 *
Eosinophiles (10^3^/uL)	0.18 ± 0.01	0.16 ± 0.02
Basophils (10^3^/uL)	0.07 ± 0.01	0.04 ± 0.02
RBC (10^6^/uL)	3.99 ± 0.02	4.56 ± 0.25 *
Hb (g/dL)	11.67 ± 0.08	14.44 ± 0.08 *
Hct (%)	36.75 ± 0.25	42.35 ± 0.21 *
MCV (fL)	92.22 ± 0.39	92.92 ± 0.38
MCH (pg)	29.32 ± 0.16	31.69 ± 0.15 *
MCHC (g/dL)	31.76 ± 0.08	34.07 ± 0.06 *
RDW-CV (%)	13.44 ± 0.12	12.54 ± 0.07 *
Platelets (10^3^/uL)	245.9 ± 3.84	273 ± 4.19 *
MPV (fL)	8.42 ± 0.12	9.10 ± 0.06 *
PCT (%)	0.20 ± 0.01	0.25 ± 0.01 *
PDW (%)	20.18 ± 0.09	15.96 ± 0.02 *
Ferritin (ng/mL)	16.10 ± 0.87	24.15 ± 0.77 *
Testosterone (ng/mL)	0.54 ± 0.02	0.61 ± 0.02 *
Estradiol (pg/mL)	1627 ± 47	1649 ± 65
Erythropoietin (mU/mL)	13.45 ± 0.91	13.77 ± 0.53
sTfR(ug/mL)	2.86 ± 0.22	1.02 ± 0.03 *
IL-6 (pg/mL)	22.53 ± 3.00	18.73 ± 1.37
Hepcidin (ng/mL)	4.35 ± 0.43	6.33 ± 0.48 *

Data are mean ± SEM. Data presented as frequencies (percentages) are assessed by the chi-square test. BMI = body mass index; WBC: white blood cells; RBC: red blood cells; Hb: hemoglobin: Hct: hematocrit; MCV: mean corpuscular volume; MCH: mean corpuscular hemoglobin; MCHC: mean corpuscular hemoglobin concentration; RDW-CV; red blood cell distribution width-coefficient of variation; MPV: mean platelet volume; PCT: plateletcrit; PDW: platelet distribution width; sTfR = soluble transferrin receptor; IL-6 = interleukin 6. * *p* < 0.01.

**Table 2:** Hematological characteristics in pregnant women studied in Lima (low altitude) according to trimester of pregnancy.

	First Trimester (N = 31)	Second Trimester (N = 52)	Third Trimester (N = 149)
Age (years)	32.01 ± 0.97 **	29.12 ± 0.97	28.8 ± 0.62 ^&&^
Gestational age (weeks)	9.29 ± 0.39 *	19.27 ± 0.59 ^#^	34.05 ± 0.25 ^&^
SpO_2_ (%)	98.2 ± 0.07	98.19 ± 0.07	98.01 ± 0.07
SBP (mm Hg)	106 ± 2.65	104 ± 1.7	102 ± 0.83
DBP (mm Hg)	72.7 ± 2.07	68 ± 1.22	70.1 ± 0.75
CaO_2_ (mL/dL)	16.48 ± 0.21 *	15.34 ± 0.23	15.53 ± 0.15 ^&^
WBC (10^3^/uL)	8.37 ± 0.38	8.94 ± 0.28	8.86 ± 0.18
Neutrophils (10^3^/uL)	5.51 ± 0.31	6.17 ± 0.24	6.21 ± 0.14 ^&&^
Lymphocytes (10^3^/uL)	2.08 ± 0.10	1.96 ± 0.07	1.86 ± 0.04 ^&&^
Monocytes (10^3^/uL)	0.50 ± 0.02	0.51 ± 0.02	0.55 ± 0.02
Eosinophiles (10^3^/uL)	0.21 ± 0.04	0.23 ± 0.03 ^##^	0.16 ± 0.01
Basophils (10^3^/uL)	0.06 ± 0.004	0.06 ± 0.004	0.07 ± 0.002
RBC (10^6^/uL)	4.17 ± 0.06 *	3.86 ± 0.05 ^##^	3.99 ± 0.03 ^&^
Hb (g/dL)	12.30 ± 0.16 *	11.46 ± 0.17	11.61 ± 0.11 ^&^
Hct (%)	38.31 ± 0.54 *	35.73 ± 0.48	36.78 ± 0.33 ^&&^
MCV (fL)	91.97 ± 0.78	92.58 ± 0.81	92.14 ± 0.53
MCH (pg)	29.56 ± 0.28	29.70 ± 0.32	29.12 ± 0.22
MCHC (g/dL)	32.15 ± 0.18	32.05 ± 0.17 ^##^	31.56 ± 0.10 ^&&^
RDW-CV (%)	12.6 ± 0.19	13.01 ± 0.21 ^#^	13.79 ± 0.17 ^&^
Platelets (10^3^/uL)	271.4 ± 9.56 **	241.3 ± 7.96	241.8 ± 4.9 ^&^
MPV (fL)	7.78 ± 0.26	8.41 ± 0.29	8.57 ± 0.14 ^&^
PCT (%)	0.21 ± 0.007	0.20 ± 0.005	0.20 ± 0.003
PDW (%)	19.49 ± 0.24	19.98 ± 0.18 ^#^	20.40 ± 0.11 ^&^
Ferritin (ng/mL)	25.49 ± 4.12	16.91 ± 1.70 ^##^	13.03 ± 0.65 ^&^
Testosterone (ng/mL)	0.73 ± 0.05	0.59 ± 0.06 ^##^	0.43 ± 0.03 ^&^
Estradiol (pg/mL)	1038 ± 131	1317 ± 78 ^#^	2028 ± 45 ^&^
Erythropoietin (mU/mL)	15.18 ± 4.10	16.35 ± 2.38	11.62 ± 0.61
sTfR(ug/mL)	2.02 ± 0.21	3.41 ± 0.68	2.84 ± 0.21 ^&^
IL-6 (pg/mL)	18.28 ± 2.17	21.63 ± 3.06	24.75 ± 5.12
Hepcidin (ng/mL)	9.67 ± 2.06 **	4.43 ± 0.56 ^#^	2.85 ± 0.39 ^&^

Data are mean ± SEM. * First vs. second trimester; ^#^ second vs. third trimester; ^&^ third vs. first trimester. *, ^#^, ^&^: *p* < 0.01; **, ^##^, ^&&^: *p* < 0.05.

**Table 3:** Hematological characteristics in pregnant women studied in Cusco (high altitude) according to trimester of pregnancy.

	First Trimester (N = 58)	Second Trimester (N = 81)	Third Trimester (N = 69)
Age (years)	27.31 ± 0.86	27.11 ± 0.71	27.31 ± 0.76
Gestational age (weeks)	9.34 ± 0.32 *	19.86 ± 0.52 ^#^	34.12 ± 0.44 ^&^
SpO_2_ (%)	95.05 ± 0.46	95.45 ± 0.26 ^#^	94.14 ± 0.26
SBP (mm Hg)	97.05 ± 1.00	97.85 ± 0.85 ^##^	101.16 ± 1.32 ^&&^
DBP (mm Hg)	62.95 ± 0.74	62.22 ± 0.63 ^##^	64.40 ± 0.83
CaO_2_ (mL/dL)	20.14 ± 0.17	20.10 ± 0.19 ^#^	18.09 ± 0.15 ^&^
WBC (10^3^/uL)	7.70 ± 0.20	7.93 ± 0.20	7.68 ± 0.18
Neutrophils (10^3^/uL)	5.30 ± 0.14	5.71 ± 0.18	5.42 ± 0.17
Lymphocytes (10^3^/uL)	1.94 ± 0.07	1.76 ± 0.06	1.90 ± 0.07
Monocytes (10^3^/uL)	0.22 ± 0.01	0.22 ± 0.01	0.23 ± 0.01
Eosinophiles (10^3^/uL)	0.22 ± 0.05	0.17 ± 0.02 ^#^	0.11 ± 0.02 ^&^
Basophils (10^3^/uL)	0.02 ± 0.001	0.02 ± 0.001	0.02 ± 0.001
RBC (10^6^/uL)	4.88 ± 0.04 *	4.43 ± 0.04	4.46 ± 0.04 ^&^
Hb (g/dL)	15.19 ± 0.14 *	14.08 ± 0.13	14.20 ± 0.11 ^&^
Hct (%)	44.31 ± 0.37 *	41.35 ± 0.34	41.83 ± 0.31 ^&^
MCV (fL)	90.87 ± 0.66 **	93.55 ± 0.70	93.87 ± 0.59 ^&^
MCH (pg)	31.16 ± 0.28	31.89 ± 0.28	31.89 ± 0.25
MCHC (g/dL)	34.27 ± 0.11	34.03 ± 0.10	33.96 ± 0.08 ^&&^
RDW-CV (%)	12.39 ± 0.14	12.68 ± 0.13	12.51 ± 0.10
Platelets (10^3^/uL)	291.88 ± 9.36	276 ± 5.7 ^##^	255.4 ± 7.11 ^&^
MPV (fL)	8.86 ± 0.10	9.01 ± 0.1 ^##^	9.42 ± 0.11 ^&^
PCT (%)	0.26 ± 0.01	0.25 ± 0.001	0.24 ± 0.01
PDW (%)	15.84 ± 0.05	15.86 ± 0.03 ^#^	16.19 ± 0.04 ^&^
Ferritin (ng/mL)	29.85 ± 1.90 *	23.72 ± 1.20 ^##^	19.89 ± 0.75 ^&^
Testosterone (ng/mL)	0.69 ± 0.06	0.67 ± 0.05 ^##^	0.49 ± 0.05 ^&&^
Estradiol (pg/mL)	968 ± 63 *	1753 ± 60 ^##^	2113 ± 156 ^&^
Erythropoietin (mU/mL)	10.84 ± 0.66 *	15.40 ± 1.12	14.45 ± 0.72 ^&^
sTfR(ug/mL)	0.90 ± 0.05	0.97 ± 0.05 ^##^	1.15 ± 0.07 ^&&^
IL-6 (pg/mL)	17.34 ± 2.87	16.51 ± 1.10	22.64 ± 3.21
Hepcidin (ng/mL)	10.01 ± 1.21 *	5.73 ± 0.67	4.00 ± 0.58 ^&^

Data are mean ± SEM. * First vs. second trimester; ^#^ second vs. third trimester; ^&^ third vs. first trimester. *, ^#^, ^&^: *p* < 0.01; **, ^##^, ^&&^: *p* < 0.05.

**Table 4:** Correlations of seven RBC parameters with four platelet parameters in 233 pregnant women at low altitude and 211 pregnant women at high altitude.

	Platelet (10^3^/uL) r-Value *p*-Value		PCT (%) r-Value *p*-Value		MPV (FL) r-Value *p*-Value		PDW (%) r-Value *p*-Value	
	LA	HA	LA	HA	LA	HA	LA	HA
RBC (10^6^/uL)	+0.05 NS	+0.23 S	+0.18 S	+0.28 S	+0.11 NS	−0.01 NS	+0.06 NS	−0.01 NS
Hb (g/dL)	−0.11 NS	−0.07 NS	−0.04 NS	−0.08 NS	+0.08 NS	+0.01 NS	+0.10 NS	+0.27 * S
HcT (%)	−0.09 NS	+0.01 NS	+0.04 NS	+0.02 NS	+0.13 NS	+0.03 NS	+0.12 NS	+0.18 S
MCV (FL)	−0.21 S	−0.30 S	−0.20 S	−0.37 S	+0.03 NS	+0.06 NS	+0.07 NS	+0.20 S
MCH (pg)	−0.28 S	−0.34 S	−0.27 S	−0.39 S	+0.03 NS	+0.03 NS	+0.03 NS	+0.14 S
MCHC (g/dL)	−0.05 NS	−0.26 S	−0.24 S	−0.35 S	−0.11 NS	−0.07 NS	−0.04 NS	−0.03 NS
RDW-CV (%)	0.28 * S	+0.16 S	0.02 S	+0.23 S	+0.13 NS	+0.07 NS	−0.08 NS	−0.14 S

Red blood cells (RBC), hemoglobin (Hb), hematocrit (Hct), mean corpuscular volume (MCV), mean corpuscular hemoglobin (MCH), mean corpuscular hemoglobin concentration (MCHC), red cell blood distribution width-coefficient of variation (RDW-CV), plateletcrit (PCT), mean platelet volume and platelet distribution width (PDW). S = significant (*p* < 0.05); NS = not significant (*p* > 0.05). * Polynomic correlation. r-value = coefficient of Pearson.

**Table 5:** Correlation of hematological parameters of the blood-red cell series, and iron status markers with gestational age (GA) in weeks in pregnant women residing at low and high altitude.

Blood Marker	GA at Low Altitude	GA at High Altitude
RBC (10^6^/uL)	−0.28 * S	−0.41 S
Hb (g(dL)	−0.23 * S	−0.33 S
Hct (%)	−0.22 * S	−0.31 S
MCV (fL)	+0.11 NS	+0.18 S
MCH (pg)	−0.14 S	+0.08 NS
MCHC (g/dL)	−0.25 S	−0.19 S
RDW-CV (%)	+0.33 * S	+0.08 NS
CaO_2_ (mL/dL)	−0.23 * S	−0.39 S
Ferritin (ng/mL)	−0.30 S	−0.36 S
sTfR (ug/nL)	+0.09 NS	+0.24 S
EPO (mU/mL)	−0.11 NS	+0.27 S
Hepcidin (ng/mL)	−0.33 ^#^ S	−0.37 ^#^ S

GA: gestational age (weeks). * Polynomic; ^#^ exponential regression. S = significant (*p* < 0.05); NS = not significant.

**Table 6:** Correlation of four RBC and platelet parameters with serum ferritin levels in pregnant women at low and at high altitude.

Hematological Marker/Serum Ferritin	Low Altitude	High Altitude
RBC (10^6^/uL)	+0.01 (NS)	+0.22 (S)
Hb (g/dL)	+0.22 (S)	+0.29 (S)
Hct (%)	+0.19 (S)	+0.26 (S)
MCV (fL)	+0.12 (NS)	+0.01 (NS)
MCH (pg)	+0.15 (S)	+0.07 (NS)
MCHC (g/dL)	+0.11 (NS)	+0.21 (S)
RDW-CV (%)	−0.30 * (S)	−0.25 * (S)
Platelets (10^3^/uL)	−0.10 (NS)	0.10 (NS)
MPV (fL)	−0.08 (NS)	−0.09 (NS)
PCT (%)	0.06 (NS)	0.05 (NS)
PDW (%)	−0.185 * (S)	−0.16 (S)

S = significant (*p* < 0.05); NS = not significant. * Power correlation.

## Data Availability

Data generated in this study are available upon reasonable request to the corresponding author.

## References

[1] Stobdan T., Akbari A., Azad P., Zhou D., Poulsen O., Appenzeller O., Gonzales G.F., Telenti A., Wong E.H.M., Saini S. (2017). New Insights into the Genetic Basis of Monge’s Disease and Adaptation to High-Altitude. Mol. Biol. Evol..

[2] Gonzales G.F., Fano D., Vásquez-Velásquez C. (2017). Necesidades de investigación para el diagnóstico de anemia en poblaciones de altura [Diagnosis of anemia in populations at high altitudes]. Rev. Peru. Med. Exp. Salud. Publica.

[3] Richalet J.P. (2021). Adaptation à l’hypoxie chronique des populations de haute altitude [Adaption to chronic hypoxaemia by populations living at high altitude]. Rev. Mal. Respir..

[4] Villafuerte F.C., Simonson T.S., Bermudez D., León-Velarde F. (2022). High-altitude erythrocytosis: Mechanisms of adaptive and mal-adaptive responses. Physiology.

[5] Costantine M.M. (2014). Physiologic and pharmacokinetic changes in pregnancy. Front. Pharmacol..

[6] Fisher A.L., Nemeth E. (2017). Iron homeostasis during pregnancy. Am. J. Clin. Nutr..

[7] Getrajdman C., Sison M., Lin H.M., Katz D. (2022). The effects of hemodilution on coagulation in term parturients: An in vitro study utilizing rotational thromboelastometry. J. Matern. Fetal Neonatal Med..

[8] Moser G., Guettler J., Forstner D., Gauster M. (2019). Maternal Platelets—Friend or Foe of the Human Placenta?. Int. J. Mol. Sci..

[9] Aguree S., Gernand A.D. (2019). Plasma volume expansion across healthy pregnancy: A systematic review and meta-analysis of longitudinal studies. BMC Pregnancy Childbirth.

[10] de Haas S., Ghossein-Doha C., van Kuijk S.M., van Drongelen J., Spaanderman M.E. (2017). Physiological adaptation of maternal plasma volume during pregnancy: A systematic review and meta-analysis. Ultrasound Obstet. Gynecol..

[11] Kumar D., Kasukurti P., Murthy S. (2017). Erythrocytes and Platelets: A Critical Analysis of their Ontogenic Relationship through Automated Parameters. J. Clin. Diagn. Res..

[12] Villamonte-Calanche W., Lam-Figueroa N., Jerí-Palomino M., De-La-Torre C., Villamonte-Jerí A.A. (2020). Maternal Altitude-Corrected Hemoglobin and at Term Neonatal Anthropometry at 3400 m of Altitude. High Alt. Med. Biol..

[13] Wu L., Sun R., Liu Y., Liu Z., Chen H., Shen S., Wei Y., Deng G. (2022). High hemoglobin level is a risk factor for maternal and fetal outcomes of pregnancy in Chinese women: A retrospective cohort study. BMC Pregnancy Childbirth.

[14] Steer P.J. (2000). Maternal hemoglobin concentration and birth weight. Am. J. Clin. Nutr..

[15] Cho J.I., Basnyat B., Jeong C., Di Rienzo A., Childs G., Craig S.R., Sun J., Beall C.M. (2017). Ethnically Tibetan women in Nepal with low hemoglobin concentration have better reproductive outcomes. Evol. Med. Public Health.

[16] Levine L.D., Gonzales G.F., Tapia V.L., Gasco M., Sammel M.D., Srinivas S.K., Ludmir J. (2015). Preterm birth risk at high altitude in Peru. Am. J. Obstet. Gynecol..

[17] Alarcón-Yaquetto D.E., Figueroa-Mujica R., Valverde-Bruffau V., Vásquez-Velásquez C., Sánchez-Huamán J.J., Jimenez-Troncoso L., Rozas-Gamarra R., Gonzales G.F. (2022). Hematological Parameters and Iron Status in Adult Men and Women Using Altitude Adjusted and Unadjusted Hemoglobin Values for Anemia Diagnosis in Cusco, Peru (3400 MASL). Physiologia.

[18] Kadikoylu G., Yavasoglu I., Bolaman Z., Senturk T. (2006). Platelet parameters in women with iron deficiency anemia. J. Natl. Med. Assoc..

[19] Campbell M.J., Julious S.A., Altman D.G. (1995). Estimating sample sizes for binary, ordered categorical, and continuous outcomes in two group comparisons. BMJ.

[20] Martin D.S., Cobb A., Meale P., Mitchell K., Edsell M., Mythen M.G., Grocott M.P., Xtreme Alps Research Group (2015). Systemic oxygen extraction during exercise at high altitude. Br. J. Anaesth..

[21] Moraga F.A., Osorio J., Jiménez D., Calderón-Jofré R., Moraga D. (2019). Aerobic Capacity, Lactate Concentration, and Work Assessment During Maximum Exercise at Sea Level and High Altitude in Miners Exposed to Chronic Intermittent Hypobaric Hypoxia (3800 m). Front. Physiol..

[22] Viruez-Soto A., Jiménez-Torres F., Sirpa-Choquehuanca V., Casas-Mamani R., Cala-Cahuay J., Maceda A., Vera-Carrasco O. (2021). Gasometría arterial en embarazo a muy alta altitud. Cuad. Hosp. Clínicas.

[23] McAuliffe F., Kametas N., Krampl E., Ernsting J., Nicolaides K. (2001). Blood gases in pregnancy at sea level and at high altitude. BJOG.

[24] Amare Y.E., Haile D. (2020). Evaluation of Pulmonary Function Tests among Pregnant Women of Different Trimesters in Debre Berhan Referral Hospital, Shoa, Ethiopia. Int. J. Womens Health.

[25] Vargas M., Vargas E., Julian C.G., Armaza J.F., Rodriguez A., Tellez W., Niermeyer S., Wilson M., Parra E., Shriver M. (2007). Determinants of blood oxygenation during pregnancy in Andean and European residents of high altitude. Am. J. Physiol. Regul. Integr. Comp. Physiol..

[26] Richlin S., Cusick W., Sullivan C., Dildy G., Belfort M. (1998). Normative oxygen saturation values for pregnant women at sea level. Prim. Care Update OB/GYNS.

[27] Bailey B., Euser A.G., Bol K.A., Julian C.G., Moore L.G. (2022). High-altitude residence alters blood-pressure course and increases hypertensive disorders of pregnancy. J. Matern. Fetal Neonatal Med..

[28] Grant I.D., Giussani D.A., Aiken C.E. (2021). Blood pressure and hypertensive disorders of pregnancy at high altitude: A systematic review and meta-analysis. Am. J. Obstet. Gynecol. MFM.

[29] Alkhaldy H.Y., Awan Z.A., Abouzaid A.A., Elbahaie H.M., Al Amoudi S.M., Andarawi M., Shehata S.F. (2020). The Prevalence of Isolated Neutropenia at High Altitude in Southern Saudi Arabia: Does Altitude Affect Leucocyte Count?. Int. J. Gen. Med..

[30] Bakrim S., Motiaa Y., Ouarour A., Masrar A. (2018). Hematological parameters of the blood count in a healthy population of pregnant women in the Northwest of Morocco (Tetouan-M’diq-Fnideq provinces). Pan Afr. Med. J..

[31] Purohit G., Shah T., Harsoda J.M. (2015). Hematological profile of normal pregnant women in Western India. Sch. J. App. Med. Sci..

[32] Hsu W.Y., Wu C.H., Hsieh C.T., Lo H.C., Lin J.S., Kao M.D. (2013). Low body weight gain, low white blood cell count and high serum ferritin as markers of poor nutrition and increased risk for preterm delivery. Asia Pac. J. Clin. Nutr..

[33] Grant I.D., Giussani D.A., Aiken C.E. (2022). Fetal growth and spontaneous preterm birth in high-altitude pregnancy: A systematic review, meta-analysis, and meta-regression. Int. J. Gynaecol. Obstet..

[34] He Z.Z., Ma S.Q., Deng L., Wang H., Li X.H., Xu Y. (2021). Microcirculation characteristics and humoral factors of healthy people from different populations at high altitude (4100 m). Sheng Li Xue Bao.

[35] Alarcón-Yaquetto D.E., Caballero L., Gonzales G.F. (2017). Association between Plasma N-Acylethanolamides and High Hemoglobin Concentration in Southern Peruvian Highlanders. High Alt. Med. Biol..

[36] Gonzales G.F., Steenland K., Tapia V. (2009). Maternal hemoglobin level and fetal outcome at low and high altitudes. Am. J. Physiol. Regul. Integr. Comp. Physiol..

[37] Umar Z., Rasool M., Asif M., Karim S., Malik A., Mushtaq G., Kamal M.A., Mansoor A. (2015). Evaluation of hemoglobin concentration in pregnancy and correlation with different altitude: A study from balochistan plateau of Pakistan. Open Biochem. J..

[38] Li A., Yang S., Zhang J., Qiao R. (2017). Establishment of reference intervals for complete blood count parameters during normal pregnancy in Beijing. J. Clin. Lab. Anal..

[39] Calis P., Karcaaltincaba D., Isik G., Buyuktaskin F., Bayram M., Karabacak O. (2020). A cross-sectional study in non-anaemic pregnant women in Turkey to assess necessity of iron supplementation. East Mediterr. Health J..

[40] Beutler E., West C. (2005). Hematologic differences between African-Americans and whites: The roles of iron deficiency and alpha-thalassemia on hemoglobin levels and mean corpuscular volume. Blood.

[41] Crispin P.J., Sethna F., Andriolo K. (2019). Red Cell and Reticulocyte Parameters for the Detection of Iron Deficiency in Pregnancy. Clin. Lab..

[42] Paliogiannis P., Zinellu A., Mangoni A.A., Capobianco G., Dessole S., Cherchi P.L., Carru C. (2018). Red blood cell distribution width in pregnancy: A systematic review. Biochem. Med..

[43] Sonaglioni A., Esposito V., Caruso C., Nicolosi G.L., Bianchi S., Lombardo M., Gensini G.F., Ambrosio G. (2020). Association between neutrophil to lymphocyte ratio and carotid artery wall thickness in healthy pregnant women. Eur. J. Obstet. Gynecol. Reprod. Biol..

[44] Amah-Tariah F.S., Ojeka S.O., Dapper D.V. (2011). Haematological values in pregnant women in Port Harcourt, Nigeria II: Serum iron and transferrin, total and unsaturated iron binding capacity and some red cell and platelet indices. Niger. J. Physiol. Sci..

[45] Wang Y., Huang X., Yang W., Zeng Q. (2022). Platelets and High-Altitude Exposure: A Meta-Analysis. High Alt. Med. Biol..

[46] Gonzales G.F., Tapia V., Vásquez-Velásquez C. (2021). Changes in hemoglobin levels with age and altitude in preschool-aged children in Peru: The assessment of two individual-based national databases. Ann. N. Y. Acad. Sci..

[47] Gassmann M., Mairbäurl H., Livshits L., Seide S., Hackbusch M., Malczyk M., Kraut S., Gassmann N.N., Weissmann N., Muckenthaler M.U. (2019). The increase in hemoglobin concentration with altitude varies among human populations. Ann. N. Y. Acad. Sci..

[48] Walle M., Asrie F., Gelaw Y., Getaneh Z. (2022). The role of platelet parameters for the diagnosis of preeclampsia among pregnant women attending at the University of Gondar Comprehensive Specialized Hospital antenatal care unit, Gondar, Ethiopia. J. Clin. Lab. Anal..

[49] Sileshi B., Urgessa F., Wordofa M. (2021). A comparative study of hematological parameters between hypertensive and normotensive individuals in Harar, eastern Ethiopia. PLoS ONE.

[50] Forstner D., Guettler J., Gauster M. (2021). Changes in Maternal Platelet Physiology during Gestation and Their Interaction with Trophoblasts. Int. J. Mol. Sci..

[51] Wiwanitkit V. (2004). Plateletcrit, mean platelet volume, platelet distribution width: Its expected values and correlation with parallel red blood cell parameters. Clin. Appl. Thromb. Hemost..

[52] McMullin M.F., White R., Lappin T., Reeves J., MacKenzie G. (2003). Haemoglobin during pregnancy: Relationship to erythropoietin and haematinic status. Eur. J. Haematol..

[53] Milman N., Graudal N., Nielsen O.J., Agger A.O. (1997). Serum erythropoietin during normal pregnancy: Relationship to hemoglobin and iron status markers and impact of iron supplementation in a longitudinal, placebo-controlled study on 118 women. Int. J. Hematol..

[54] Delaney K.M., Guillet R., Pressman E.K., Ganz T., Nemeth E., O’Brien K.O. (2021). Serum Erythroferrone During Pregnancy Is Related to Erythropoietin but Does Not Predict the Risk of Anemia. J. Nutr..

[55] Wolfson G.H., Vargas E., Browne V.A., Moore L.G., Julian C.G. (2017). Erythropoietin and Soluble Erythropoietin Receptor: A Role for Maternal Vascular Adaptation to High-Altitude Pregnancy. J. Clin. Endocrinol. Metab..

[56] Luo Q., Zhao H., Jiang Y., Guo J., Lv N., Tang J., Li S., Zhang D., Bai R., Chen G. (2020). Association of blood metal exposure with testosterone and hemoglobin: A cross-sectional study in Hangzhou Birth Cohort Study. Environ. Int..

[57] Gonzales G.F., Chaupis D. (2015). Higher androgen bioactivity is associated with excessive erythrocytosis and chronic mountain sickness in Andean Highlanders: A review. Andrologia.

[58] Firestein M.R., Romeo R.D., Winstead H., Goldman D.A., Grobman W.A., Haas D., Mercer B., Parker C., Parry S., Reddy U. (2022). Elevated prenatal maternal sex hormones, but not placental aromatase, are associated with child neurodevelopment. Horm. Behav..

[59] Bitsko R.H., Holbrook J.R., O’Masta B., Maher B., Cerles A., Saadeh K., Mahmooth Z., MacMillan L.M., Rush M., Kaminski J.W. (2022). A Systematic Review and Meta-analysis of Prenatal, Birth, and Postnatal Factors Associated with Attention-Deficit/Hyperactivity Disorder in Children. Prev. Sci..

[60] Dybdahl M., Dalgård C., Glintborg D., Andersen M.S., Christesen H.T. (2022). Maternal Testosterone Concentrations in Third Trimester and Offspring Handgrip Strength at 5 Years: Odense Child Cohort. J. Clin. Endocrinol. Metab..

[61] Charles S.M., Julian C.G., Vargas E., Moore L.G. (2014). Higher estrogen levels during pregnancy in Andean than European residents of high altitude suggest differences in aromatase activity. J. Clin. Endocrinol. Metab..

[62] Tiwari M., Kotwal J., Kotwal A., Mishra P., Dutta V., Chopra S. (2013). Correlation of haemoglobin and red cell indices with serum ferritin in Indian women in second and third trimester of pregnancy. Med. J. Armed. Forces India.

[63] Kim S., Cho S.Y. (2018). Investigation of Iron Metabolism for Regulating Megakaryopoiesis and Platelet Count According to the Mechanisms of Anemia. Clin. Lab..

[64] Brissot E., Troadec M.B., Loréal O., Brissot P. (2021). Iron and platelets: A subtle, under-recognized relationship. Am. J. Hematol..

[65] Park M.J., Park P.W., Seo Y.H., Kim K.H., Park S.H., Jeong J.H., Ahn J.Y. (2013). The relationship between iron parameters and platelet parameters in women with iron deficiency anemia and thrombocytosis. Platelets.

[66] Huamaní C., Sarmiento W., Cordova-Heredia G., Cruz-Huanca L., Damian-Saavedra P., Antonio D. (2022). Prediction of Blood Viscosity Based on Usual Hematological Parameters in a Clinically Healthy Population Living in a High-Altitude City. High Alt. Med. Biol..

[67] Julian C.G., Wilson M.J., Lopez M., Yamashiro H., Tellez W., Rodriguez A., Bigham A.W., Shriver M.D., Rodriguez C., Vargas E. (2009). Augmented uterine artery blood flow and oxygen delivery protect Andeans from altitude-associated reductions in fetal growth. Am. J. Physiol. Regul. Integr. Comp. Physiol..

[68] Browne V.A., Julian C.G., Toledo-Jaldin L., Cioffi-Ragan D., Vargas E., Moore L.G. (2015). Uterine artery blood flow, fetal hypoxia and fetal growth. Philos. Trans. R. Soc. Lond. B Biol. Sci..

[69] Viteri F.E., Casanueva E., Tolentino M.C., Díaz-Francés J., Erazo A.B. (2012). Antenatal iron supplements consumed daily produce oxidative stress in contrast to weekly supplementation in Mexican non-anemic women. Reprod. Toxicol..

[70] Casanueva E., Viteri F.E. (2003). Iron and oxidative stress in pregnancy. J. Nutr..

